# Human Sapovirus among Outpatients with Acute Gastroenteritis in Spain: A One-Year Study

**DOI:** 10.3390/v11020144

**Published:** 2019-02-08

**Authors:** Miguel F. Varela, Enrique Rivadulla, Alberto Lema, Jesús L. Romalde

**Affiliations:** Department of Microbiology and Parasitology, CIBUS-Faculty of Biology, Universidade de Santiago de Compostela, 15782 Santiago de Compostela, Spain; miguel.fernandez.varela@usc.es (M.F.V.); enrique.rivadulla@usc.es (E.R.); alberto.lema.blanco@usc.es (A.L.)

**Keywords:** sapovirus, gastroenteritis, RT-qPCR, genotyping

## Abstract

Viral agents of human gastroenteritis affect people of all ages across the globe. As a mainly self-limiting disease, it is difficult to evaluate the real prevalence of etiological agents circulating in each region. Many of the analyzed outbreaks are caused by viruses of the family Caliciviridae, especially the genus *Norovirus* (NoV). Most studies have focused on other enteric viruses, leaving sapovirus (SaV) underestimated as an important emerging human threat. This one-year study analyzed clinical samples from hospital outpatients with acute gastroenteritis in Spain, with the aim of revealing the importance of human SaV as an emerging viral pathogen. A total of 2667 stools were tested using reverse transcription (RT)-qPCR to detect and quantify SaV. Sapovirus was detected in all age groups, especially in infants, children, and the elderly. The prevalence was 15.64% (417/2667), and was slightly higher in 0–2- and 3–5-year-olds (19.53% and 17.95%, respectively) and much lower in 13–18-year-olds (9.86%). Positive samples were detected throughout the year, with peaks of detection during autumn and the late winter to early spring months. The mean value for the quantified samples was 6.5 × 10^5^ genome copies per gram of stool (GC/g) (range 2.4 × 10^3^–6.6 × 10^11^ GC/g). RT-nested PCR and sequencing were used for further genotyping. Genetic characterization showed a predominance of genogroup I (GI), followed by GII and GIV. The detection of multiple genotypes suggests the circulation of different strains without any clear tendency. The results obtained suggest SaV as the second major gastroenteritis agent after NoV in the region.

## 1. Introduction

Foodborne diseases have been, during the last years, the eighth major cause of human deaths, becoming third on the African continent, and have been the cause of 9% of mortalities in children worldwide, according to the Global Health Observatory [1]. Parasites, bacteria, and viruses are the infectious agents causing the thousands of deaths every year associated with these illnesses. Enteric viruses are among the major contributors, including a wide range of etiological agents such as adenovirus (AdV), astrovirus, rotavirus (RV), aichivirus, sapovirus (SaV), hepatitis A and E virus, and norovirus (NoV). Most of them are considered foodborne viruses, but their transmission occurs not only via the ingestion of infected food and water (the irrigation of fruits and vegetables, and shellfish harvested in polluted waters), but also via person-to-person contact (infected workers and handlers), contaminated environments, and fomites. They are characterized by low infectious doses and high levels of viral excretion [2].

The genus *Sapovirus*, from the family Caliciviridae, has been recently included into the Contaminant Candidate List (CCL4) by the U.S. Environmental Protection Agency as an emerging microbial agent producing mild gastrointestinal illness [3]. SaV are classified into five genogroups, four of which (I, II, IV, V) infect humans, even though the existence of several additional genogroups infecting different animals has been suggested [4,5]. The morphology corresponds to a non-enveloped 30–38 nm diameter icosahedral virus, with single-stranded positive-sense RNA and a genome containing two or three open reading frames, depending on the genogroup [4].

Outbreaks happen throughout the year in all age groups in different settings, such as in childcare centers, schools, colleges, hospitals, restaurants, hotels, and cruises. SaV transmission occurs by the cited fecal–oral route and, similar to NoV, may have a very low infectious dose [4]. The incubation period lasts from less than a day until four days [6]. Patients present acute diarrhea (with excretion of 10^5^ to 10^11^ SaV genome copies per gram of stool (GC/g)) and vomiting as major symptoms, although abdominal pain, chills, and headaches have also been reported [7]. Illness is usually self-limiting, with therapy just for dehydration and electrolyte imbalances, although some individuals may require hospitalization [7]. Shedding levels persist on high even several weeks after (more than 10^5^ GC/g) [8], and no or limited long-term immunity results from infection, meaning that one person may be repeatedly infected. In addition, asymptomatic cases have been reported [9].

The main purpose of the study was to determine the presence of human SaV circulating in the Spanish population in the Galician region over one year, taking into account an age group classification as well as the drifts in time of the different genotypes.

## 2. Materials and Methods

### 2.1. Sampling

The study comprised stool samples from a total of 2667 outpatients suffering from acute gastroenteritis treated at the University Hospital of A Coruña city, in Spain, which serves more than half a million people. Samples were divided by age group as follows: 0–2 (*n* = 886), 3–5 (*n* = 195), 6–12 (*n* = 244), 13–18 (*n* = 71), 19–59 (*n* = 653), ≥60 (*n* = 597), and unspecified age (*n* = 21). Sampling was carried out from July 2010 to June 2011, and between 31 and 112 stool samples were received weekly at the laboratory (58 on average). Coinfection analysis was performed with previously obtained data for RV, AdV, and bacterial pathogens detected at the hospital, and for human NoV genogroups I (GI) and II (GII) at our laboratory [10]. This study was carried out in accordance with the Declaration of Helsinki, as revised in 2000. This non-interventional study included no additional procedures. Anonymized biological material was obtained only for standard viral diagnosis. The Spanish Biomedical Research Law (14/2007; article 3i) does not require written informed consent for such a protocol.

### 2.2. Viral RNA Extraction

The samples employed were previously processed, and viral RNA was extracted during the cited study [10]. Briefly, they were diluted at 10% (*w*/*v*) in supplemented phosphate-buffered saline (PBS), shaken, and centrifuged for viral recovery. RNA extraction was carried out using a NucleoSpin^®^ RNA Virus kit (Macherey-Nagel, Düren, Germany) and by adding known amounts of Mengovirus clone vMC0 (MG) as an extraction efficiency control. In the case of a low volume of original extracted RNA or of low extraction efficiencies, viral RNA was re-extracted from the original stool sample homogenates and stored in our facilities at −80 °C.

### 2.3. Reverse Transcription (RT)-qPCR Methodology

Detection and quantification methodologies were based on the principles established in the latest ISO procedures for enteric viruses [11,12]. The protocol described by Oka et al. [13] was employed with minor modifications. Briefly, reverse primer SaV1245R (5’-CCCTCCATYTCAAACACTA-3´), forward primer SaV124F (5´-GAYCASGCTCTCGCYACCTAC-3´), and TaqMan probe SaV124TP (5´-FAM-CCRCCTATRAACCA-MGB-NFQ-3´) were utilized under the following thermal conditions: Reverse transcription at 50 °C for 20 min, denaturation at 95 °C for 15 min, continued by 45 amplification cycles with denaturation at 95 °C for 15 s and annealing and elongation at 50 °C for 1 min. RT-qPCR was conducted using a Platinum Quantitative RT-PCR ThermoScript One-Step System kit (Invitrogen, Carlsbat, CA, USA) in an MX3000P QPCR System thermocycler (Stratagene, SanDiego, CA, USA) in 96-well plates.

For all the samples, a dilution of 1:10 was also tested to reduce the effect of potential polymerase inhibitors present in the matrix, and for some samples a dilution of 1:100 was necessary as well. The negative control for each assay consisted of a well in the plate with nuclease-free water and the same RT-qPCR mix. The extraction efficiency control by MG consisted of a comparison between the threshold cycle value (Ct) of the MG positive control and the Ct for each analyzed sample: The efficiency was considered valid if above 5%. The presence of polymerase inhibitors and the RT-qPCR efficiency were calculated using external controls (ECs) in each assay plate, consisting of independent wells with 2.5 μL of sample and 2.5 μL SaV positive control. Ct values of the ECs were compared to those of the positive control well, and the amplification was considered valid if the efficiency was above 25%. Control materials were obtained by cloning the 112 bp target fragment of the polymerase-capsid junction (nucleotide positions 5073–5184 of GI Sapporo strain full-length genome, accession number HM002617) into pGEM-T easy plasmid, transforming *Escherichia coli* DH5a competent cells, and then purifying and quantifying using spectrophotometry (A260 nm). After this, standard curves were calculated using serial dilutions. Neither the extraction efficiency nor the RT-qPCR efficiency were used to modify the quantification values obtained in the samples. The limit of detection (LOD) was settled in 5.2 × 10^2^ GC/g, and the limit of quantification (LOQ) in 2.2 × 10^3^ GC/g.

### 2.4. Genetic Analysis

Genotyping of the detected SaV strains was carried out using an RT-nested PCR protocol that amplifies a partial fragment of the capsid gene of human genogroups [14]. RT consisted of 5 μL of positive SaV RNA template in a final volume of 20 μL mix, using a RevertAid Reverse Transcriptase kit (Thermo Scientific, USA) with reverse primers SV-R13 (5′-GGTGANAYNCCATTKTCCAT-3′) and SV-R14 (5′-GGTGAGMMYCCATTCTCCAT-3′) (1 μM) for 60 min at 42 °C. First-round PCR was conducted using 5 μL of cDNA in a final volume of 50 μL, with the same reverse primers and forward primers, SaV124F, SaV1F (5′-TTGGCCCTCGCCACCTAC-3′), and SaV5F (5′-TTTGAACAAGCTGTGGCATGCTAC-3′) (all 0.5 μM). Amplification conditions were 94 °C for 2 min denaturation, then 40 cycles of 94 °C for 30 s denaturation, 50 °C for 30 s for primer annealing, 72 °C for 2 min for extension, and finally 72 °C for 10 min for late extension. Second-round PCR was performed for 45 cycles under similar conditions (extension 1 min) with primers SV-R2 (5′-GWGGGRTCAACMCCWGGTGG-3′) and 1245Rfwd (5′- TAGTGTTTGARATGGAGGG-3′) (0.5 μM) and 5 μL from the first-round PCR product. Both amplifications were performed with a Fermentas DreamTaq DNA Polymerase kit (ThermoScientific, Waltham, MA, USA). Nested-PCR products were visualized in 2% agarose electrophoresis gels, and amplicons approximately 420 bp in size were considered for purification and sent to be genotyped.

The obtained sequences were aligned and analyzed with software packages Lasergene 7 (DNASTAR Inc., Madison, WI, USA) and MEGA version 6 [15]. Neighbor-joining (NJ) phylogenetic dendrograms were constructed with software MEGA version 6 using a Kimura two-parameter model (bootstrap of 1000 replicates). SaV reference strains were retrieved from GenBank, and the strains obtained in this study are also available at GenBank under accession numbers LT985022-LT985081, LT985914-LT985948, and LT986236-LT986326.

### 2.5. Statistics

Statistical analysis was conducted with R Statistical Software version 3.4.3 (R-Project, Vienna, Austria). Analytical experimental data on detection frequencies were analyzed through a chi-square test to determine if the SaV prevalence was dependent on age. In the same way, quantification results were converted into a logarithmic format and tested with a one-way analysis of variance (ANOVA) to study any relationship between the SaV viral loads and the age groups [16].

## 3. Results

### 3.1. Prevalence

**Figure 1:**
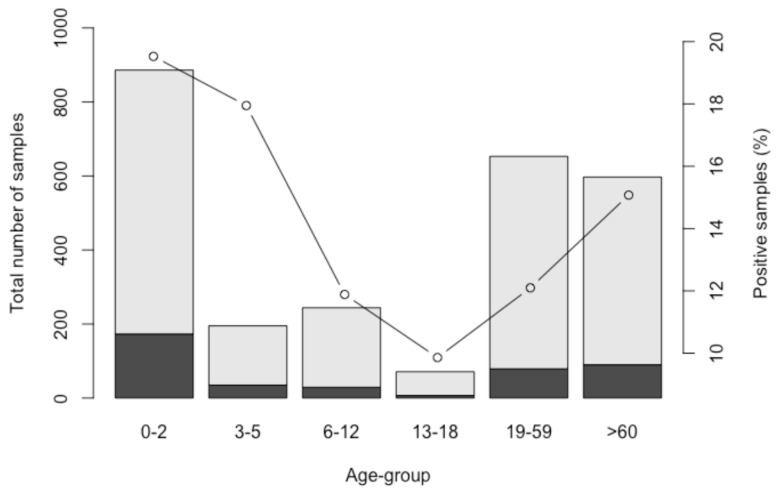
Sapovirus (SaV)-negative (light gray) and -positive samples (black), as well as human SaV prevalence within the different age groups.

Extraction and RT-qPCR efficiencies were classified as valid in all cases (>5% and >25%, respectively). Molecular assays were performed in the 2667 stool samples, and human SaV was detected in 417 (15.64%) of them. The results obtained showed that human SaV was present in all age groups, with a higher incidence in infants under 2 years old (173/886, 19.5%) and in children 3–5 years old (35/195, 17.9%). The rest of the age groups rendered as follows: 6–12 (29/244, 11.9%), 13–18 (7/71, 9.9%), 19–59 (79/653, 12.1%), and ≥60 (90/597, 15.1%). Statistical analysis with a chi-squared test showed a significant association of SaV detection with age group (*p* = 5.9 × 10^−4^). Prevalence decreased from childhood to the adult years, and it rose again in elders above 60 years old (Figure 1).

### 3.2. Coinfection

**Figure 2:**
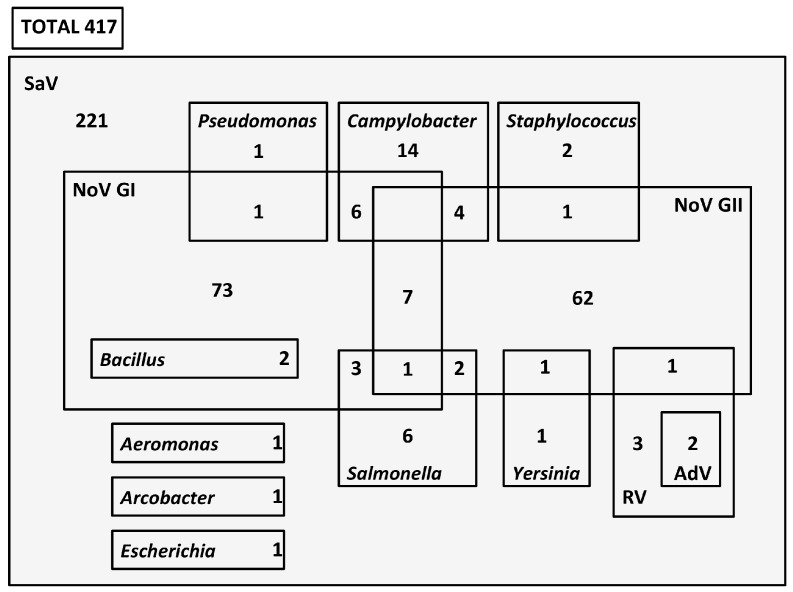
Diagram showing the number of SaV coinfections with other viral and bacteriological agents. Data for other pathogens obtained from a previous study [10].

From the 417 total SaV positive samples, 196 presented at least another gastroenteric microbial pathogen (Figure 2). SaV showed coinfection with NoV in 164 cases, of which 85 were with genogroup I (GI), 71 with genogroup II (GII), and 8 with both genogroups. Of those 85 SaV–NoV GI detections, 6 samples were positive for *Campylobacter* spp., 3 samples for *Salmonella* spp., 2 samples for *Bacillus cereus*, and 1 sample presented *Pseudomonas aeruginosa*, too. Among those other 71 SaV–NoV GII-positive samples, bacterial pathogens *Staphylococcus aureus* and *Yersinia enterocolitica* were present in 1 patient for each one, and *Salmonella* spp. in another 2 cases. On the other hand, some pediatric cases were also previously analyzed at the hospital for RV and AdV (total of 125), and 3 coinfections were detected with SaV–RV, 2 with SaV–RV–AdV, and 1 with SaV–RV–NoV GII (Figure 2).

When analyzing these results, taking into account the age group classification, it was observed that in the 0–2 age group, all the SaV–RV coinfections (including SaV–RV–AdV) occurred, as well as most of the detected coinfections with *Campylobacter* spp. and *Salmonella* spp. (Supplementary Materials, Figure S1A). Apart from that, the combined presence of SaV with NoV GI and *Bacillus cereus* was only detected in the 19–59 age group, and other different coinfections with *Pseudomonas aeruginosa*, *Aeromonas* spp., or *E. coli* 0157:H7 occurred just in the ≥60-year-old age group (Supplementary Materials, Figure S1E,F).

With the previous and actual analysis gathered for single detections and coinfections, the possible etiology of the illness could be elucidated for 47.4% of the examined gastroenteritis cases included in this study.

### 3.3. Seasonality

**Figure 3:**
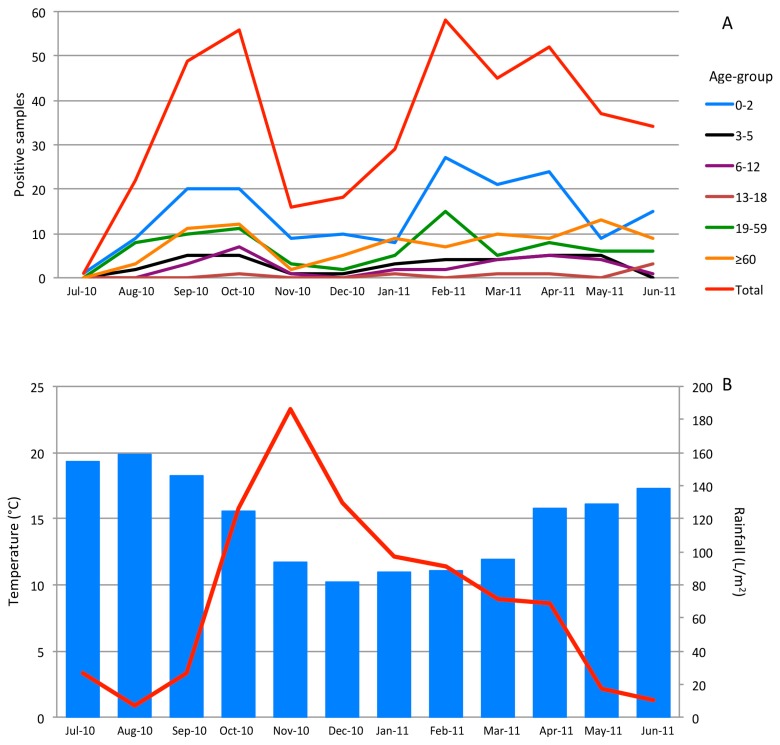
(**A**) Positive samples in the different age groups during the study. (**B**) Monthly rainfall (L/m^2^) (line) and environmental temperatures (degrees Celsius) (bars), obtained from www.meteogalicia.es.

The highest number of SaV detections was not observed during the coldest and rainiest months of the study, but in early autumn and late winter to early spring (Figure 3). September, October, and February showed important prevalence percentages (21.6%, 20.4%, and 21.1%, respectively), and prevalence kept high until spring. All age groups followed this pattern, although prevalence in the ≥60 group presented an increasing tendency from November (Figure 3A).

### 3.4. Quantification

**Figure 4:**
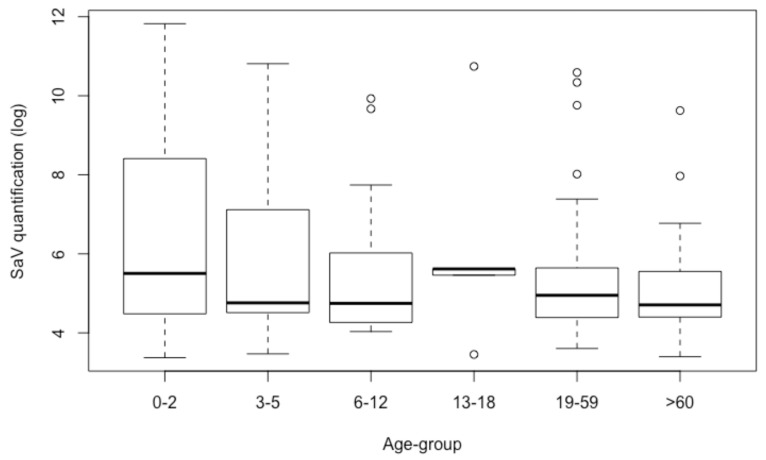
Boxplot showing logarithmic SaV quantifications for each age group.

A total of 129 positive samples ranged below the LOQ (2.2 × 10^3^ GC/g). The other 288 cases rendered an overall mean value of 6.5 × 10^5^ GC/g, with a minimum value of 2.4 × 10^3^ GC/g and a maximum of 6.6 × 10^11^ GC/g (Figure 4). Variations among viral loads in the age groups were significant when analyzed by ANOVA (*p* = 2.0 × 10^−4^). The age group between 0 and 2 presented the highest quantification, and elders (≥60 years old) the lowest. When comparing the age groups of infants and children to the elderly, the differences in quantification were 1.5 log units between 0 and 2 and ≥60 (*p* = 1.4 × 10^−7^), and 1.0 log unit between 3 and 5 and ≥60 (*p* = 0.037) (Figure 4).

### 3.5. Genetic Characterization

**Figure 5:**
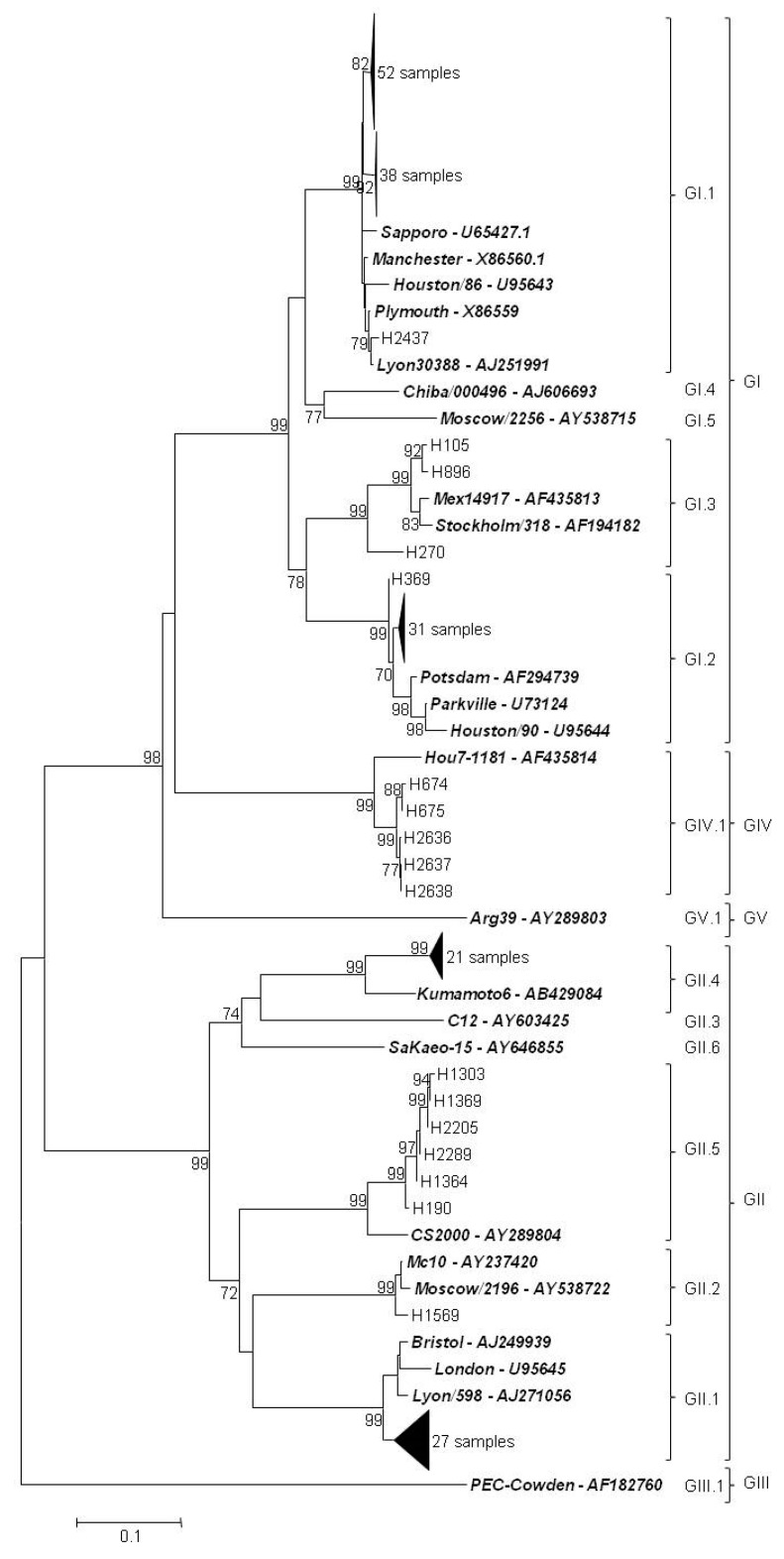
Phylogenetic tree based on the partial capsid gene sequences of SaV using the neighbor-joining algorithm. Bootstrap values (greater than 70%) are shown at each node as percentages of 1000 replicates. Reference sequences are in boldface italics. Porcine enteric calicivirus (Cowden) classified as GIII was used as outgroup. GenBank accession numbers of the reference strains used are detailed in the tree. Bar: Nucleotide substitutions per site.

Sequences suitable for genetic characterization were obtained for 186 positive samples. Of them, 126 samples corresponded to genogroup I, 55 samples to genogroup II, and 5 samples to genogroup IV (Figure 5; Supplementary Materials, Figure S2). Genogroup I sequences were classified into GI.1 (*n* = 91) (the most predominant genotype), GI.2 (*n* = 32), and GI.3 (*n* = 3). On the other side, genogroup II sequences were split into genotypes GII.1 and GII.4 (*n* = 27 and *n* = 21, respectively), genotype GII.5 (*n* = 6), and GII.2 (*n* = 1).

**Figure 6:**
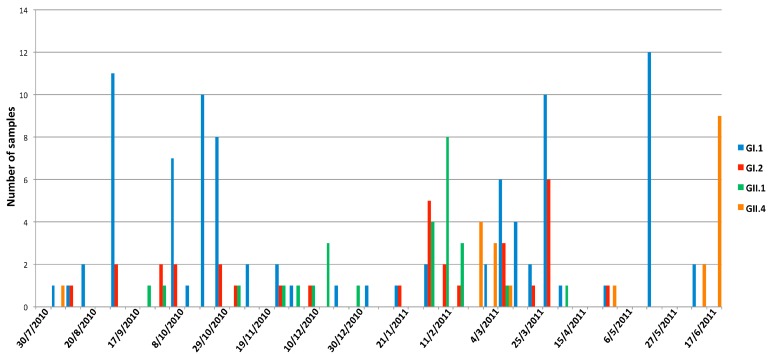
Weekly temporal distribution of the most prevalent SaV genotypes.

The temporal distribution of the different genotypes showed a predominance of GI.1 during the first quarter of the study, approximately from July until the end of October, followed by a decrease in the overall detection during late autumn to early winter. Later, the general diversity increased with detections of genotypes GI.2 and GII.1 and the appearance of GII.4. At the end of the study, only GI.1 and GII.4 remained as dominant genotypes (Figure 6).

Although GI.3 sequences (*n* = 3) were only detected in infants 0–2 and children 3–5, and genogroup IV cases did not occur during childhood periods (Table 1), no clear tendency was observed for the most prevalent genotypes, as GI.1, GI.2, GII.1, and GII.4 sequences were present in all groups.

Short-term upturns of certain genotypes, namely GI.1 and GII.4, were observed throughout the study. The samples in each of these groups showed 100% nucleotide identity, which could indicate the appearance of SaV outbreaks in the population (Supplementary Materials, Figure S2). However, the linkage among cases or the existence of a common viral vehicle could not be confirmed due to the lack of epidemiological data.

## 4. Discussion

The present study provides novel data about the role of SaV in patients with an acute gastroenteritis diagnosis receiving health care in Spain. Putting together our results with those from a previous study [10], from the 2667 cases, etiology was established in 47.4% of them, including both bacterial and viral agents.

Most of the SaV long-term studies worldwide have shown prevalence rates ranging from 2.2% to 12.7% [4], and many have been located, as in this research, in developed countries such as Japan, the U.S., or the U.K. [17,18,19]. The present study showed slightly higher SaV prevalence (15.6%), more in line with the 17% positive rate found in Nicaragua [20].

Moreover, patients 0–2 and 3–5 years old presented the highest positive rates, which was in accordance with the established notion of higher prevalence of this pathogen in children [19,21]. Elder people ≥60 presented 15.1% prevalence, demonstrating that together with infants, they are considered to be the most vulnerable population against viral acute gastroenteritis [22].

Mixed infections in patients could lead to more severe gastrointestinal symptoms, and therefore, analyses of clinical cases for more than one viral agent are needed. In 164 samples (39.3%), NoV and SaV were simultaneously detected, which was a much higher level than what can be found in the literature, where mixed infections with both caliciviruses do not usually exceed 10% of cases [17,18,19,20]. NoV–SaV mixed infections in the present study could be explained by the elevated prevalence of SaV, compared to other surveys. Pediatric coinfections with RV are scarce [18,19], and only occurred in six cases in this study, three of which showed triple mixed infections: Two with SaV–RV–AdV and one with SaV–RV–NoV.

Regarding seasonality, SaV was detected throughout the entire period, with higher incidence in September, October, and from February to May, in line with other studies [23,24,25], where its prevalence was high during spring months. Our results were in concordance with the normally accepted hypothesis that SaV is mostly detected in the cold and rainy seasons [26,27]. Observing the results previously obtained [10], the highest prevalence rates of NoV cases occurred between November and January, when SaV prevalence was at its lowest: Moreover, NoV prevalence rates decreased during the mentioned SaV high peaks of abundance (data not shown). Since, to our knowledge, no other work about SaV prevalence on a weekly basis has been reported, it is difficult to confirm if this alternance between both viruses has been detected in other geographic regions. However, a possible similar trend could be inferred from the studies of Pongsuwanna et al. [28] and Shioda et al. [29] in Thailand and Kenya, respectively.

The mean value of the quantified samples was 6.5 × 10^5^ GC/g of stool, which was in accordance with most of the studies providing information about shedding levels [8]. These levels gradually decreased after onset of illness, and therefore, based on the detection of mixed infection, some low viral load quantification cases (also under LOQ) could be, hypothetically, a combination of late SaV and early coinfection with other pathogens, which could cause severe symptoms of acute gastroenteritis with hospital care requirements.

Genetic analysis is crucial to elucidate the current circulating SaV in the population regionally and worldwide. Investigations have revealed that GI and GII are the most common SaV genogroups [30]. Genotype I.1 is the most common genotype around the globe, and GI.2 has been increasingly detected in many European countries [31], and also in northern Africa [32]. The results from this research support those from previous studies, as GI.1 and GI.2 were the two most abundant SaV genotypes (91 and 32 cases, respectively), followed by GII.1. It is noteworthy to point out the detection of some sequences belonging to rare SaV genotypes [33], including GII.4 (21 sequences), GII.5 (6 sequences), and GI.3 (3 sequences). Genotype GII.4 SaV was also recently detected in Thailand [34]. On the other hand, GIV was indeed a genotype normally detected at high rates in developed countries around 2007 [4], but not so much in this study. Environmental studies conducted in Spain have obtained findings that mirror what was observed in this study, showing a rich variety of sequences circulating, especially genotypes GI.1 and GI.2 [14,35,36].

Surveillance systems in hospitals and healthcare centers are mostly limited to detecting bacterial pathogens. Since many emerging enteric viruses produce mild and often uninvestigated infections, their recognition as possible critical human pathogens has been underestimated. Most of them are difficult to study and have symptoms similar to those caused by bacterial pathogens. This study should help to position human SaV, at least, as one of the foremost causes of nonbacterial acute gastroenteritis agents in Spain, especially in some age groups.

## Figures and Tables

**Table 1:** Genetic distribution of SaV in the different age groups (years old).

Genotype	0–2	3–5	6–12	13–18	19–59	≥60	Unknown	Total
GI.1	41	8	8	1	14	19		91
GI.2	11	5	4		5	5	2	32
GI.3	1	2						3
GII.1	17	1	1		6	2		27
GII.2		0			1			1
GII.4	7	1	1	1	5	6		21
GII.5	2	1			1	2		6
GIV				1	2	2		5
Total	79	18	14	3	34	36	2	186

## Supplementary Materials

The following are available online at http://www.mdpi.com/1999-4915/11/2/144/s1, Figure S1: Coinfection cases of SaV with other viral and bacteriological agents for the different age groups analyzed; Figure S2: Similarity matrices obtained by MegAlign of the sequence analysis for each of the genotype upturns.
