# Supplementary material for: Human Sapovirus among Outpatients with Acute Gastroenteritis in Spain: A One-Year Study
Source: Viruses. 2019 Feb 8;11(2):144. doi: 10.3390/v11020144 (PMC6409837; doi:10.3390/v11020144)
Supplement: Supplementary file 1 [file viruses-11-00144-s001.pdf]

Human sapovirus among outpatients with acute gastroenteritis in Spain: a one year study

Miguel F Varela, Enrique Rivadulla, Alberto Lema, Jesús L Romalde

Department of Microbiology and Parasitology, CIBUS-Faculty of Biology, Universidade de Santiago de Compostela. 15782, Santiago de Compostela, Spain

Supplementary Material

Supplementary Figure S1. Each of the 6 images (A-F) shows the number of coinfections cases of SaV with other viral and bacteriological agents for the different age-group analysed. Data for other pathogens obtained from previous study [16].

A

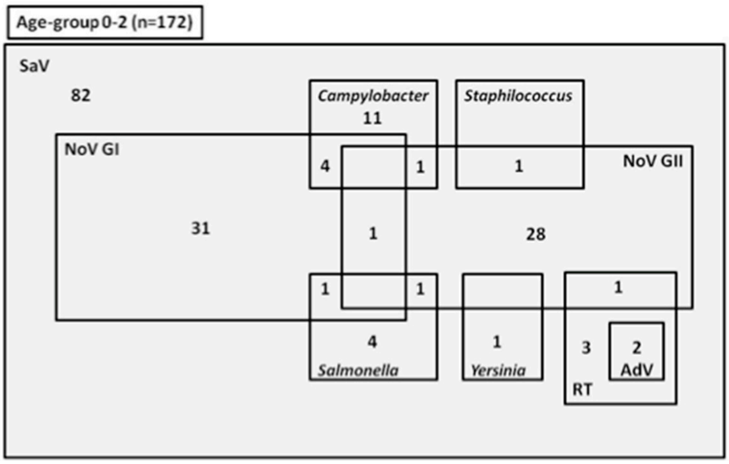

B

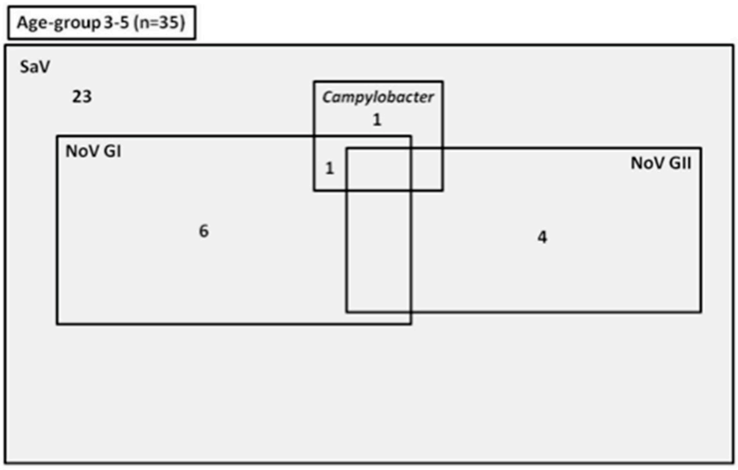

C

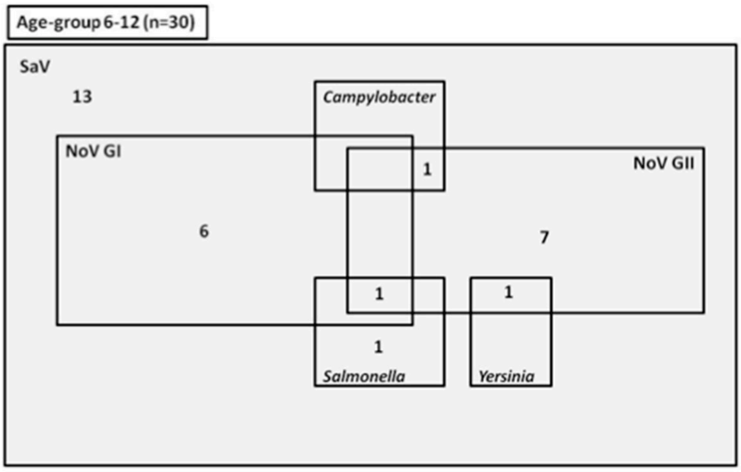

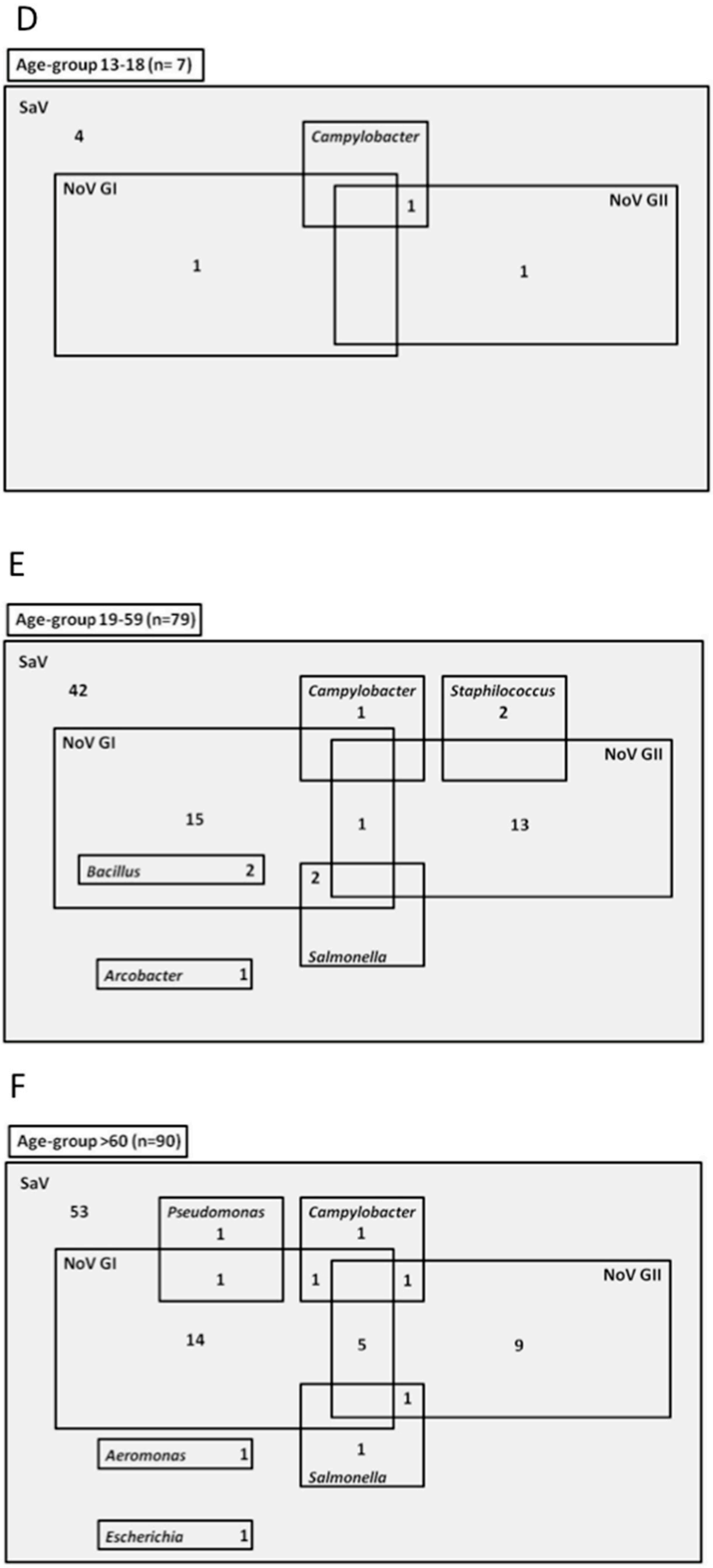
D
Age-group 13-18 (n= 7)
SaV
4
Campylobacter
NoV GI
1
NoV GII
1
1
E
Age-group 19-59 (n=79)
SaV
42
Campylobacter
1
Staphilococcus
2
NoV GI
NoV GII
15
1
13
Bacillus 2
2
Salmonella
Arcobacter 1
F
Age-group >60 (n=90)
SaV
53
Pseudomonas
1
Campylobacter
1
NoV GI
1
1
1
NoV GII
14
5
9
1
Aeromonas 1
1
Salmonella
Escherichia 1

Supplementary Figure S3. Phylogenetic trees based on the partial capsid gene sequences of SaV using the Neighbor-Joining algorithm for the sequences obtained in the present study belonging to genotype GI.2 (A), GI.1 (B), GII.1 (C) and GII.4 (D). Bootstrap values (greater than 70 %) are shown at each node as percentages of 1,000 replicates. Bar, nucleotide substitutions per site.

A

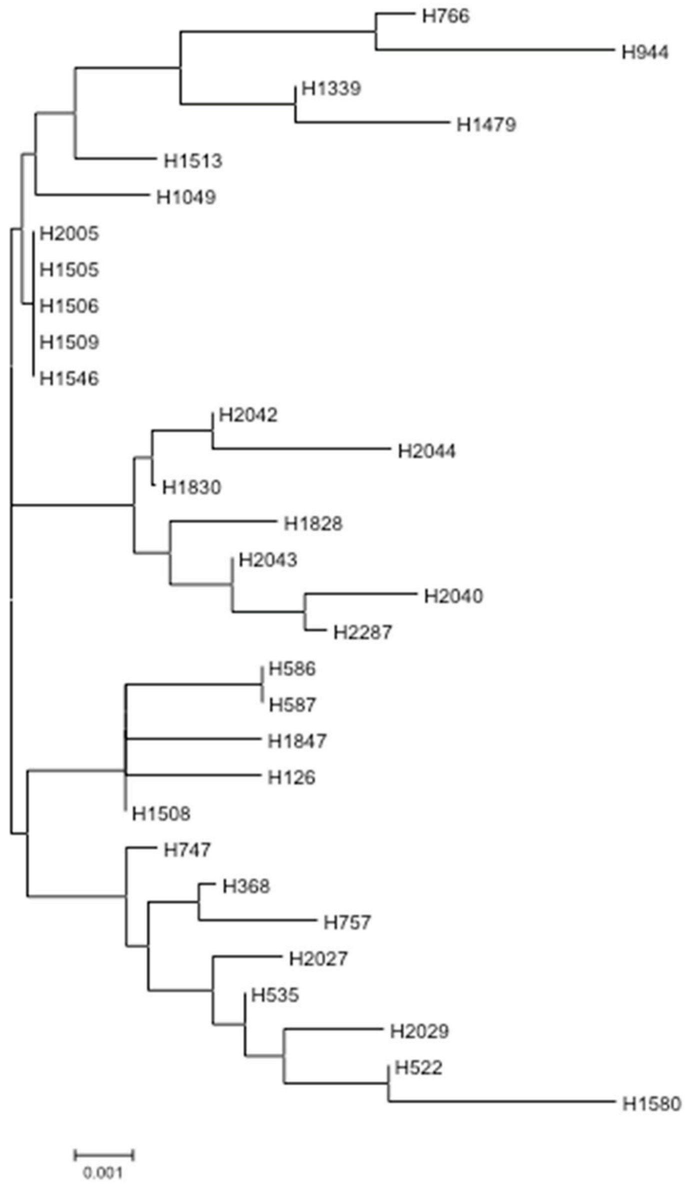

B

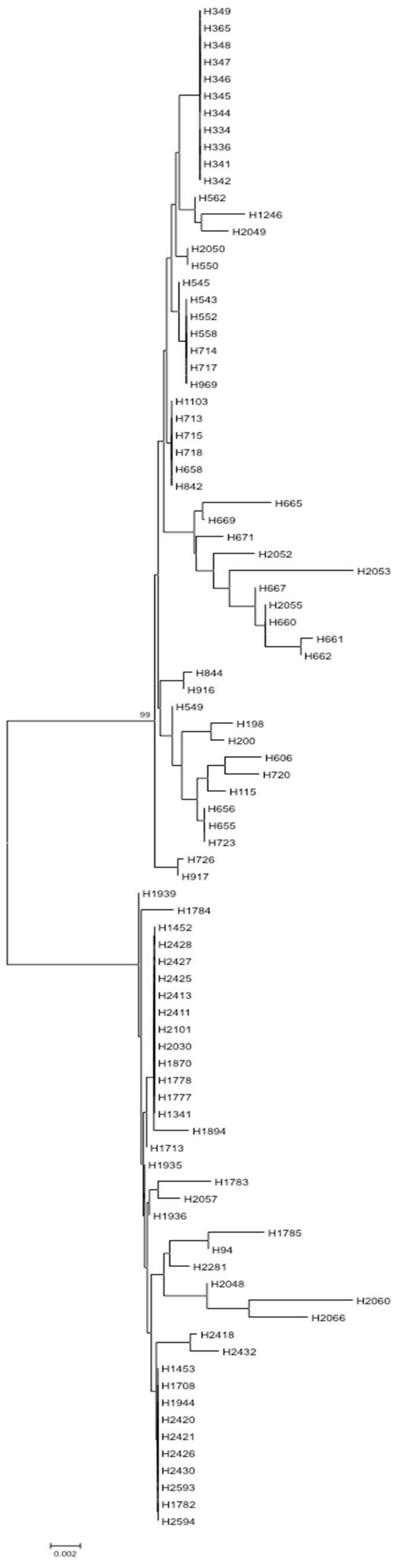
H349
H365
H348
H347
H346
H345
H344
H334
H336
H341
H342
H562
H1246
H2049
H2050
H550
H545
H543
H552
H558
H714
H717
H969
H1103
H713
H715
H718
H658
H842
H665
H669
H671
H2052
H2053
H667
H2055
H660
H661
H662
H844
H916
99
H549
H198
H200
H606
H720
H115
H656
H655
H723
H726
H917
H1939
H1784
H1452
H2428
H2427
H2425
H2413
H2411
H2101
H2030
H1870
H1778
H1777
H1341
H1894
H1713
H1935
H1783
H2057
H1936
H1785
H94
H2281
H2048
H2060
H2066
H2418
H2432
H1453
H1708
H1944
H2420
H2421
H2426
H2430
H2593
H1782
H2594
0.002

C

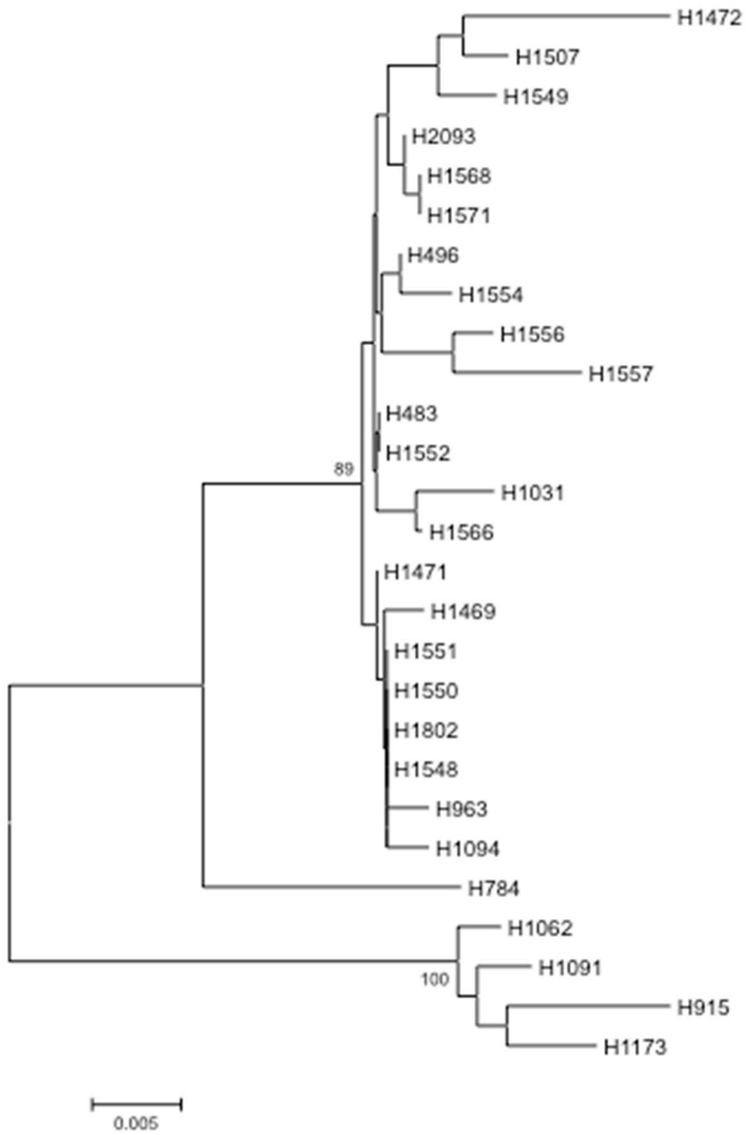
H1472
H1507
H1549
H2093
H1568
H1571
H496
H1554
H1556
H1557
H483
H1552
89
H1031
H1566
H1471
H1469
H1551
H1550
H1802
H1548
H963
H1094
H784
H1062
H1091
100
H915
H1173
0.005

D

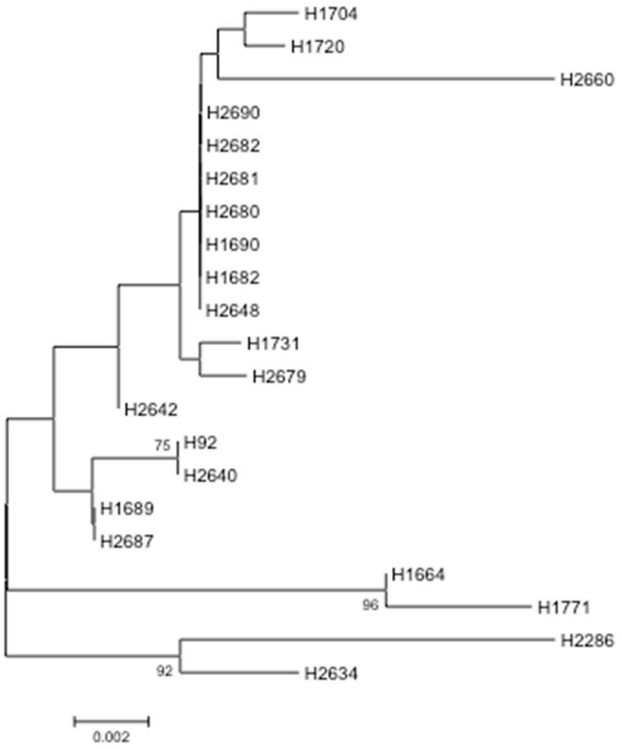
H1704
H1720
H2660
H2690
H2682
H2681
H2680
H1690
H1682
H2648
H1731
H2679
H2642
75
H92
H2640
H1689
H2687
H1664
96
H1771
H2286
92
H2634
0.002
